# Assessing the effects of the first 2 years of industry‐led badger culling in England on the incidence of bovine tuberculosis in cattle in 2013–2015

**DOI:** 10.1002/ece3.3254

**Published:** 2017-08-04

**Authors:** Lucy A. Brunton, Christl A. Donnelly, Heather O'Connor, Alison Prosser, Stuart Ashfield, Adam Ashton, Paul Upton, Andrew Mitchell, Anthony V. Goodchild, Jessica E. Parry, Sara H. Downs

**Affiliations:** ^1^ Department of Epidemiological Sciences Animal and Plant Health Agency Addlestone UK; ^2^ Department of Infectious Disease Epidemiology Faculty of Medicine School of Public Health Imperial College London MRC Centre for Outbreak Analysis and Modelling London UK

**Keywords:** badgers, bovine tuberculosis, cattle, culling, wildlife

## Abstract

Culling badgers to control the transmission of bovine tuberculosis (TB) between this wildlife reservoir and cattle has been widely debated. Industry‐led culling began in Somerset and Gloucestershire between August and November 2013 to reduce local badger populations. Industry‐led culling is not designed to be a randomized and controlled trial of the impact of culling on cattle incidence. Nevertheless, it is important to monitor the effects of the culling and, taking the study limitations into account, perform a cautious evaluation of the impacts. A standardized method for selecting areas matched to culling areas in factors found to affect cattle TB risk has been developed to evaluate the impact of badger culling on cattle TB incidence. The association between cattle TB incidence and badger culling in the first 2 years has been assessed. Descriptive analyses without controlling for confounding showed no association between culling and TB incidence for Somerset, or for either of the buffer areas for the first 2 years since culling began. A weak association was observed in Gloucestershire for Year 1 only. Multivariable analysis adjusting for confounding factors showed that reductions in TB incidence were associated with culling in the first 2 years in both the Somerset and Gloucestershire intervention areas when compared to areas with no culling (incidence rate ratio (IRR): 0.79, 95% CI: 0.72–0.87, *p* < .001 and IRR: 0.42, 95% CI: 0.34–0.51, *p* < .001, respectively). An increase in incidence was associated with culling in the 2‐km buffer surrounding the Somerset intervention area (IRR: 1.38, 95% CI: 1.09–1.75, *p* = .008), but not in Gloucestershire (IRR: 0.91, 95% CI: 0.77–1.07, *p* = .243). As only 2 intervention areas with 2 years of data are available for analysis, and the biological cause–effect relationship behind the statistical associations is difficult to determine, it would be unwise to use these findings to develop generalizable inferences about the effectiveness of the policy at present.

## 1 INTRODUCTION

Bovine tuberculosis (TB) caused by the bacterium *Mycobacterium bovis* is an important problem for the British cattle industry. Existing controls include testing and slaughter of test‐positive cattle, with herd test frequency determined by local incidence, along with surveillance of all slaughtered cattle in the abattoir. But despite these controls, the incidence of TB in cattle in England has shown a generally increasing trend over the last thirty years. In 2015, around 5% of cattle herds were under movement restriction due to a TB incident in England, and in the high risk area of England, this rose to around 11% (Harris et al., 2017).

European badgers (*Meles meles*) are a host species for *M. bovis* and represent a wildlife reservoir of infection. The use of culling to control the transmission of *M. bovis* between this wildlife reservoir and cattle has been widely debated. There is evidence from the Randomized Badger Culling Trial (RBCT) that proactive (systematic and widespread) culling of badgers for at least 4 years could reduce the incidence of confirmed TB in cattle by 23.2% (95% CI: 12.4%–32.7%) (Donnelly et al., 2006, 2007). The RBCT was conducted in England between 1998 and 2007. The net effect of proactive culling per year in the RBCT was initially detrimental as a 24.5% increase in incidence (95% CI: 0.6% lower to 56.0% higher) was observed in a 2‐km‐wide buffer around the culled area (Donnelly et al., 2007). Detrimental effects were attributed to the disruption of badger social structures (perturbation) affecting contact rates between cattle and badgers (Woodroffe et al., 2006). However, an overall benefit was observed after the third year of culling and subsequent culls (Donnelly et al., 2007).

The first round of industry‐led culling took place in two areas, one in west Somerset and the other in west Gloucestershire between August and November 2013 (hereafter referred to as the Somerset intervention area and Gloucestershire intervention area). Culling licenses were issued for the two areas by Natural England (an executive nondepartmental public body advising Defra on the natural environment) under the Protection of Badgers Act 1992 to enable groups of farmers and landowners to reduce local badger populations. Licenses were subject to a number of criteria. These included the application area to be at least 150 km^2^, at least 70% of the land to be accessible for culling, cattle herds to be subject to annual TB testing and “reasonable biosecurity” to be in place (Defra 2015). It was also a requirement that the culling should plan to reduce the estimated badger population by 70% and be conducted for a minimum of 4 years (Defra, 2015). The aim of the initial culls was to assess the practicalities and impact of the intervention on badger population density. Using a combination of cage trapping and controlled shooting, 1,296 badgers were culled in the Somerset intervention area in Years 1 and 2 combined and 1,198 were culled in the Gloucestershire intervention area in the 2 years combined. The target minimum number that was estimated to result in a 70% reduction in the badger population was not achieved in either area in Year 1 and was achieved in the Somerset intervention area only in Year 2, although there is uncertainty around the calculation of these minimum numbers due to difficulties in estimating the true badger population size (AHVLA 2014, Defra 2014c,d). The third year of culling took place in both areas during autumn 2015. A further area was licensed in Dorset in 2015, and seven new areas were licensed in 2016. Data for analyzing cattle TB incidence in the year following the culls in 2015 were not available for this analysis.

Our aim was to measure and assess any association between the badger culling intervention and the incidence of TB in local cattle, taking account of the methodological constraints resulting from this not being a study based on sound intervention study design principles. TB incidence in cattle herds located within areas where industry‐led culling is conducted (“intervention areas”) is compared with TB incidence in herds in unculled areas matched on characteristics that affect cattle TB risk (“comparison areas”) in a similar, but not identical, approach to that used for analyzing the impact of culling during the RBCT (Donnelly et al., 2003, 2006, 2007). The null hypothesis being tested is that TB incidence is the same in the intervention areas and their comparison areas in the years since badger culling began. Cattle TB incidence is also monitored among herds in 2‐km “buffer areas” surrounding the intervention areas and compared to incidence among herds in similarly defined areas around unculled comparison areas to monitor for potentially adverse effects on cattle TB incidence (Donnelly et al., 2006). The definition of incidence being used for these analyses is the incidence of OTF‐W (Officially Tuberculosis Free status—Withdrawn) incidents. OTF‐W incidents are those where confirmatory evidence of *M. bovis* infection has been identified in at least one bovine animal slaughtered for disease control purposes. They also include other incidents upgraded to OTF‐W for epidemiological reasons and slaughterhouse case‐disclosed incidents that are confirmed through culture of *M. bovis*.

Here we report an unadjusted analysis of OTF‐W incidence rate per 100 herd years at risk for the first 2 years following industry‐led culling, alongside more detailed analysis with adjustment for other factors that are likely to be associated with a risk of TB in cattle.

## 2 METHODS

### 2.1 Selection of comparison areas

Areas were selected from within England which met the following inclusion criteria: within a high‐risk TB area (Defra 2014a); cattle herds subject to annual TB testing; having no land closer than 2 km to any intervention area boundary (at the time of selection). Boundary information for the intervention areas was used in an automated programming procedure using ArcGIS 10.0 software (ESRI Release 10.0. Redlands, CA, USA) to generate a population of potential comparison areas with centroids shifted 5 km from one another and rotated at 45‐degree intervals. Comparison areas were selected in May 2014, around 9 months after the start of the culls in late 2013 without any consideration of cattle TB incidence data after the culls began. They were matched to intervention areas on factors known to affect cattle TB incidence in an attempt to control for confounding factors that could explain between‐area variation not attributable to culling and therefore bias the analysis. These are described in more detail below.

### 2.2 Ranking of comparison areas for similarity to an intervention area

Each intervention area and potential comparison area was described by six factors or attributes previously associated with TB incidence that had been extracted from the APHA Sam surveillance database (Table 1). The distribution of each factor was summarized using deciles, and the absolute difference between the decile to which the intervention area belonged and the decile to which each potential comparison area belonged was calculated. A score based on the sum of the absolute differences for each of the attributes was then used to rank potential comparison areas, with areas having the smallest summed absolute difference ranked highest. No weighting was applied to the ranking attributes.

**Table 1:** Distribution of attributes used to rank comparison areas across intervention areas and matched comparison areas (as mean values across 10 comparison areas)

Area	All TB incidents prior to baseline date^a^		OTF‐W incidents prior to baseline date		Number of herds	Herd size median	Distance to intervention area km	RBCT proactive area %
	1 year	3 year	1 year	3 year				
Somerset								
Intervention central	30	105	27	84	154	53.5	0	0.5
Intervention buffer^b^	16	43	13	36	88	39.5	0	3.4
Comparison central	31.4	92.1	25.8	74.4	186.3	56.3	62.1	0.03
Comparison buffer	20.1	56.3	16.8	44.7	119.9	61.7	62.1	2.4
Gloucestershire								
Intervention central	17	90	15	69	215	46.0	0	0
Intervention buffer	22	55	16	41	121	48.0	0	0
Comparison central	27.8	83.2	23.3	66.6	171.3	46.2	33.3	0.7
Comparison buffer	18.1	53.8	15.2	43.6	100.8	52.8	33.3	5.5

a TB incidents include all incidents. Only OTF‐W incidents were used to rank comparison areas.

b Lower values for some variables because buffer area to the north extends into the sea.

### 2.3 Selection of matched comparison areas based on rank

The highest ranked comparison areas were preferentially selected using a semi‐automated process that also sequentially excluded comparison areas that covered part of a higher ranking area or one of the two intervention areas. Ten comparison areas were selected for each intervention area, in order to minimize the consequences of future censoring of comparison areas due to initiation of new intervention areas that cover land in a previously selected comparison area. The process of ranking potential comparison areas and for manually selecting highest‐ranking comparison areas was conducted independently by two members of the project team, and any differences in outputs were reconciled. Other attributes likely to be associated with cattle TB incidence were summarized for each intervention and comparison area (Table 2).

**Table 2:** Distribution of explanatory factors across intervention areas and matched comparison areas (as mean values across 10 comparison areas)

Area	Dairy herds %^a^	Estimated badger main sett density mean/km^2^ a	Number of badgers removed historically			Flood zone 3%^a^	Motorway total length/km^a^	Urban area %^a^	Number of farms^a^	>1 Fragment in area %^a^	All land inside area %^a^	All land inside central or buffer areas %^a^
			1972–1990	1990–1998	1999–2006							
Somerset												
Intervention central	8.4	0.409	0	375	230	16.2	0	0	153	83.7	65.4	81.0
Intervention buffer	3.4	0.387	0	87	102	3.4	0	0	87	75.9	37.9	56.3
Comparison central	20.3	0.557	154	101	3	9.8	3.5	3.3	185	79.0	58.1	74.2
Comparison buffer	21.5	0.550	64	69	17	7.2	2.4	4.3	119	76.9	31.1	53.3
Gloucestershire												
Intervention central	17.7	0.626	52	366	62	16.2	0	0	214	83.6	67.3	78.5
Intervention buffer	19.8	0.657	21	97	47	14.0	38.1	8.6	119	71.4	31.1	51.3
Comparison central	16.4	0.589	175	88	28	7.3	4.1	5.3	169	77.6	54.9	71.8
Comparison buffer	15.7	0.578	138	80	61	8.7	5.8	4.4	100	74.4	29.0	50.8

a On baseline date. Baseline dates for Somerset and Gloucestershire were 26 August 2013 and 03 September 2013, respectively.

### 2.4 Statistical analysis

The observed OTF‐W incidence rate in cattle herds was calculated for each of the two intervention areas, in the 2 km‐wide unculled buffer area around each intervention area, and in the 20 comparison areas (10 per intervention area) and their 2 km‐wide buffers for the first and second years following the baseline date (the date on which the first cull started) and the periods 0–12 months, 12–24 months, and 24–36 months prior to the baseline date. In each case, the herd years‐at‐risk was calculated using results from whole herd tests as the sum of the time herds in each area were unrestricted and therefore at risk of new infection (a new OTF‐W incident) during the period of interest (Downs et al., 2013). Crude OTF‐W incidence rate ratios (IRRs) were calculated for both the central intervention areas and buffer areas in each reporting period. 95% confidence intervals were calculated, and a probability level of *p* < .05 was considered to be statistically significant.

For the adjusted analysis, data for explanatory variables were merged with the OTF‐W incidence event data and the herd years‐at‐risk denominator data. Adjusted IRRs were estimated using Poisson regression models comparing intervention with comparison areas separately for the central areas (i.e., the intervention or comparison areas) and the buffer areas (the 2‐km buffer areas surrounding intervention and comparison areas), controlling for factors known to be associated with TB incidence. Initial models contained a single intervention status variable which represented the badger culling intervention in both the Somerset and Gloucestershire intervention areas combined and a variable representing the geographical area (coded as Somerset or Gloucestershire) which included both the intervention areas and their matched comparison areas. The inclusion of a variable representing the interaction between the intervention status and area was also explored in these initial models.

Two models were built, to estimate the effect of intervention on OTF‐W incidence in the central areas (Model A) and the buffer areas (Model B) in the first 2 years of culling. Each model included two separate intervention variables, one for each of the Somerset and Gloucestershire intervention areas in order estimate the independent effects of the intervention in each geographical area. Prior to building the models, bivariable associations between the outcome and explanatory variables were assessed, and pairwise correlations were explored to investigate any colinearity between explanatory variables. Explanatory variables considered to be important potential confounders a priori were included in the initial models. These consisted of the variables used to match the comparison areas—area (Somerset or Gloucestershire), historic (3‐year) incidence, median herd size, the proportion of herds that were dairy, distance to the matched intervention area and the proportion of the land in the area subjected to proactive culling during the RBCT—to account for imperfect matching, as well as estimated badger sett density (Judge, Wilson, Macarthur, Delahay, & McDonald, 2014). Additional factors were then added to the models one at a time to assess the effect they had on the estimated impact of the intervention. Akaike's Information Criterion (AIC) was used to select models with good fit and parsimonious use of explanatory variables (as recommended by Burnham & Anderson, 2002).

Count and continuous explanatory variables included historic incidence, herd size, herd years at risk in the first 2 years of culling, and the number of badgers removed as part of historical control operations including the RBCT and earlier government‐led culling. These were transformed by adding 0.5 if any values were zero and taking the natural logarithm. Incidence rate ratios were estimated for all variables as the exponent of the estimated Poisson regression coefficient. The herd years‐at‐risk variable represented the time herds in an area were classified to be free of *M. bovis* infection based on TB testing information. To account for any overdispersion (greater‐than‐expected variance between areas due to clustering of cattle TB incidents), the “robust” or “sandwich” estimator of variance (Huber, 1967; White, 1982) was used to ensure that confidence intervals were adequately adjusted. Overdispersion in the final models was then assessed using the deviance and Pearson statistics.

A sensitivity analysis of the influence of individual comparison areas on the estimated associations between industry‐led culling and OTF‐W incidence was conducted by systematically removing one area at a time from the analysis. A further sensitivity analysis was conducted by systematically removing one explanatory variable at a time from the models. The aim of this was to identify which of the explanatory variables most influenced the estimated associations between industry‐led culling and OTF‐W incidence.

As part of our validation of the final models, three further sets of models were developed. The first set examined OTF‐W incidence in the first 2 years since culling began, but using a reduced number of explanatory variables (area, time at risk, and historic incidence) in an approach more similar to the analyses of RBCT data (Donnelly et al., 2003, 2006). The second set examined OTF‐W incidence in the year prior to the culls to explore whether there was any association between the intervention areas and incidence prior to the start of culling; and the third set estimated effects for the individual years since culling began. The results of these validation analyses are presented in the 1, 2, 3.

## 3 RESULTS

### 3.1 Comparison of characteristics in intervention areas and matched comparison areas

The characteristics of the intervention areas and buffers and their matched comparison areas are presented in Table 1. There were 6,658 and 6,387 potential comparison areas generated through the 5‐km shifts, respectively, for the Somerset and Gloucestershire intervention areas. The 10 selected comparison areas were fairly well matched to their respective intervention areas when values for the ranking factors were averaged (Table 1). The proportion of all incidents that were OTF‐W was over 80% for all comparison areas. The greatest differences between the intervention and comparison areas were in historic TB incidence and the number of herds. The numbers of OTF‐W incidents prior to baseline were slightly lower overall in Somerset comparison areas compared to the Somerset intervention area in the 3 years prior to baseline with a difference of 10 fewer incidents on average. The mean number of OTF‐W incidents in the Gloucestershire comparison areas was higher 1 year prior to baseline than the intervention area with a difference of eight more incidents. The mean number of herds in Somerset comparison areas was slightly higher overall than in the intervention area (32 more herds on average), while the mean number of herds in the Gloucestershire comparison areas overall was slightly lower than in the intervention area (44 fewer herds on average).

**Figure 1:**
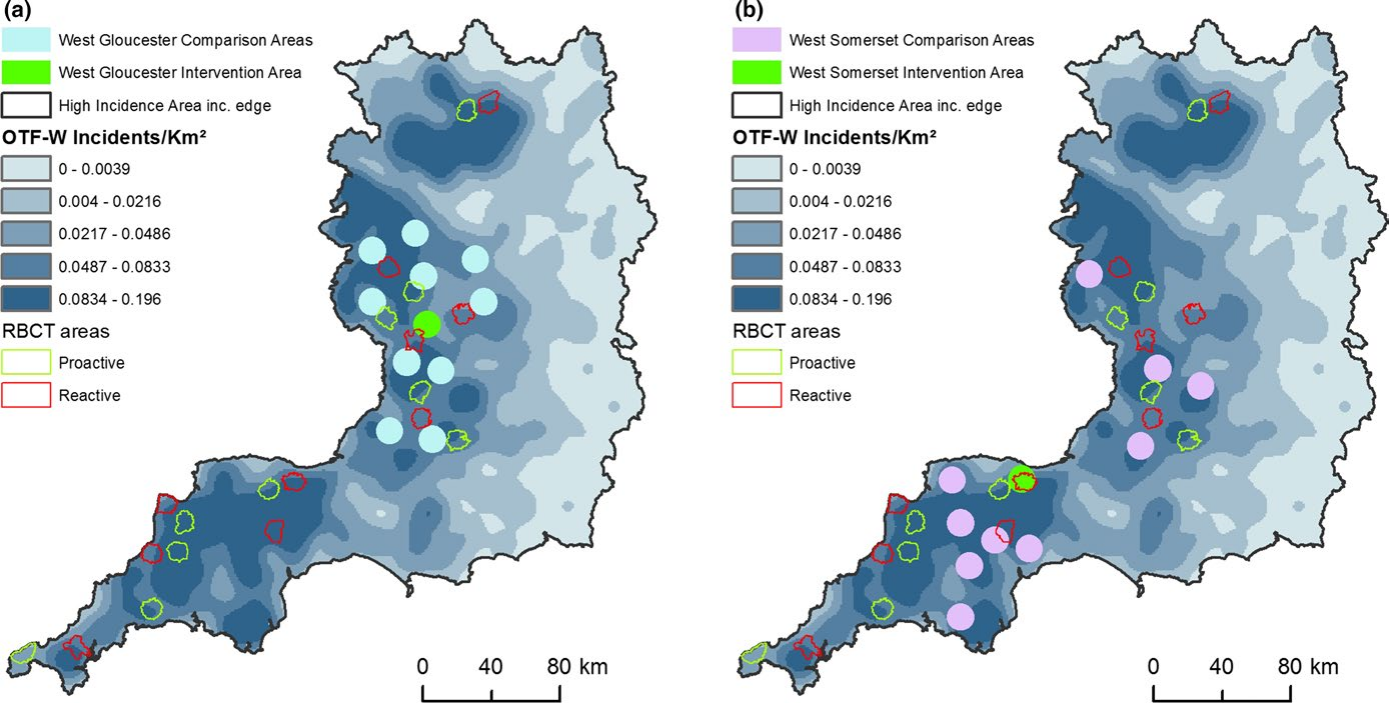
(a) Locations of Gloucestershire Intervention Area and 10 matched comparison areas across map of smoothed cattle TB herd incidence in the high incidence area of England, 2013. Culling and comparison areas are symbolized by a solid circle that approximates actual size. OTF‐W incident density was created using the spatial analyst kernel density tool within ArcGIS 10.0. (b) Locations of Somerset Intervention Area and ten matched comparison areas across map of smoothed cattle TB herd incidence in the high incidence area of England, 2013. Culling and comparison areas are symbolized by a solid circle that approximates actual size. OTF‐W incident density was created using the spatial analyst kernel density tool within ArcGIS 10.0

The geographical locations of the intervention areas and 10 matched comparison areas are shown in Fig. 1. Gloucestershire comparison areas were clustered around the Gloucestershire intervention area (mean distance 33.3 km, *SD* 15.7), whereas some of the 10 comparison areas matched to the Somerset area were further afield (mean distance 62.1 km, *SD* 32.1).

None of the matched comparison areas contained more than 4% of RBCT proactive area land (Fig. 1) although some of the comparison area buffer areas contained more RBCT proactive land (maximum of 17% for one Gloucestershire comparison area buffer). Seven Somerset comparison areas and nine Gloucestershire comparison areas had had badgers removed as part of control operations at some point in the past. The largest number of badgers removed historically in a Somerset comparison area was 1,589 and in a Gloucestershire comparison area was 1,586. Estimated badger sett density was slightly lower in the Somerset intervention area than in the matched comparison areas and similar in the Gloucestershire intervention and comparison areas (Table 2).

### 3.2 Analysis of the impact of the intervention on OTF‐W incidence

The observed OTF‐W incidence rates per 100 herd years at risk for each of the central intervention and comparison areas, and their respective buffer areas for Year 1, Year 2, and for each of the 3 years prior to the cull are presented in Table 3. Descriptive analyses without adjustment for confounding factors showed no statistically significant difference between OTF‐W incidence rates in the Somerset central intervention area and comparison areas for Year 1 or Year 2. A weak difference in incidence rate between the Gloucestershire central intervention and comparison areas was observed for Year 1 (IRR: 0.64, *p* = .05), but not for Year 2. No differences in incidence rates were observed between the intervention buffer areas and comparison buffer areas.

**Table 3:** OTF‐W incidence rates per 100 herd years at risk and unadjusted incidence rate ratios (IRRs) for central and buffer intervention areas versus the central and buffer comparison areas, respectively, in Somerset and Gloucestershire, for each 12‐month reporting period

12‐month Reporting period	Intervention central	Comparison central	IRR	95% Confidence interval		*p* value	Intervention buffer	Comparison buffer	IRR	95% Confidence interval		*p* value
Somerset												
3 years prior	19.7	15.0	1.32	0.86	1.95	.173	10.7	13.6	0.79	0.35	1.54	.503
2 years prior	21.3	13.3	1.60	1.06	2.35	.022	17.0	13.5	1.26	0.67	2.18	.407
1 year prior	23.0	15.3	1.50	0.97	2.24	.054	16.4	15.3	1.07	0.56	1.89	.778
Year 1	18.9	14.5	1.30	0.83	1.95	.209	17.1	17.3	0.99	0.53	1.70	.998
Year 2	14.0	14.4	0.97	0.58	1.53	.910	11.7	15.6	0.75	0.35	1.42	.384
Gloucestershire												
3 years prior	14.6	13.5	1.08	0.71	1.59	.683	13.0	16.2	0.81	0.44	1.38	.437
2 years prior	11.4	14.8	0.77	0.48	1.19	.231	9.4	15.0	0.62	0.29	1.18	.136
1 year prior	7.7	14.9	0.52	0.29	0.86	.006	14.0	16.9	0.83	0.46	1.39	.488
Year 1	9.5	14.8	0.64	0.39	1.02	.049	12.9	14.0	0.93	0.50	1.58	.802
Year 2	9.2	12.4	0.74	0.44	1.19	.213	12.1	14.6	0.83	0.44	1.43	.511

In the initial models for the multivariable analysis for the first 2 years of culling which contained a single intervention variable and an area variable, the inclusion of an interaction between the intervention and area generated a *p*‐value of <.001 for both the central and buffer areas. This indicated that the effects of the interventions in the two central areas should be examined separately by including area‐specific intervention variables. The final models comparing OTF‐W incidence in the first 2 years of culling, adjusted for explanatory variables, for the central areas (Model A), and the buffer areas (Model B) are presented in Table 4.

**Table 4:** Multivariable Poisson regression models describing the effect of intervention (badger culling) stratified by area on the number of OTF‐W^a^ incidents *in the first 2 years since culling began*, adjusted for explanatory variables. Model A describes the effect in the central intervention areas compared with matched central comparison areas, and Model B describes the effect in the 2‐km buffer area for the intervention areas compared with buffer areas for matched comparison areas

	IRR^b^	Robust *SE*	*p* value	95% Confidence interval	
Model A—Central areas					
Intervention effect in Somerset area^c^	0.791	0.040	<.001	0.716	0.874
Intervention effect in Gloucestershire area^c^	0.417	0.041	<.001	0.344	0.507
Area = Somerset	1.103	0.173	.532	0.811	1.501
Log transformed herd years at risk in first 2 years of culling	5.251	0.699	<.001	4.044	6.817
Log transformed OTF‐W incidence rate over 3 years prior	1.555	0.136	<.001	1.310	1.847
Log transformed median herd size	0.397	0.308	.233	0.087	1.815
Proportion of herds that are dairy	1.019	0.003	<.001	1.013	1.024
Distance to intervention (km)	0.996	0.001	<.001	0.995	0.998
Estimated badger sett density per 100 km^2^	0.995	0.003	.031	0.990	1.000
Proportion of land involved in proactive culling in the RBCT	1.003	0.026	.922	0.953	1.055
Log transformed number of badgers culled historically	1.032	0.010	.001	1.013	1.052
Proportion of farms with more than one fragment of land in the area	1.026	0.007	<.001	1.012	1.040
Model B—Buffer areas					
Intervention effect in Somerset area^c^	1.379	0.166	.008	1.090	1.746
Intervention effect in Gloucestershire area^c^	0.908	0.075	.243	0.771	1.068
Area = Somerset	1.106	0.108	.298	0.914	1.339
Log transformed herd years at risk in first 2 years of culling	1.955	0.193	<.001	1.612	2.372
Log transformed OTF‐W incidence rate over 3 years prior	2.076	0.181	<.001	1.749	2.464
Log transformed median herd size	1.056	0.145	.694	0.806	1.382
Proportion of herds that are dairy	1.019	0.009	.031	1.002	1.037
Distance to intervention (km)	0.999	0.001	.351	0.996	1.002
Estimated badger sett density per 100 km^2^	0.840	0.410	.721	0.323	2.184
Proportion of land involved in proactive culling in the RBCT	0.977	0.005	<.001	0.967	0.988
Proportion of land classed as urban	1.053	0.013	<.001	1.027	1.079
Proportion of farms with more than one fragment of land in the area	1.050	0.008	<.001	1.034	1.067

a Officially Tuberculosis Free status Withdrawn.

b Incidence rate ratio.

c Intervention is industry‐led badger culling, as described in the Section 1.

For the central areas (Model A), the number of badgers removed historically and the proportion of farms with more than one fragment of land in the area were included in the model alongside the a priori explanatory variables as they affected the association between the intervention and OTF‐W incidence in Somerset and reduced the AIC (Table 4). A lower IRR for the intervention was observed in the Somerset and Gloucestershire central areas (both *p* < .001), with a considerably lower IRR in Gloucestershire (IRR: 0.42; 95% CI: 0.34, 0.51) than in Somerset (IRR: 0.79; 95% CI: 0.72, 0.87). As expected, increases in time at risk and historic incidence were significantly associated with increased OTF‐W incidence. Associations with increased OTF‐W incidence were also observed for the proportion of herds that were dairy, the total number of badgers removed historically, and the proportion of farms with more than one fragment of land in the area. Increases in the distance to the intervention area and the estimated number of badger setts per 100 km^2^ were both associated with a decrease in OTF‐W incidence in the first 2 years of culling. The Deviance and Pearson goodness‐of‐fit tests for overdispersion (*p* = .898 and *p* = .904, respectively) indicated no evidence of there being significantly more variation in the data than expected.

The sensitivity analysis of OTF‐W incidence in the central areas showed that with the removal of one of the Somerset comparison areas (WS02), there was no longer an association between the intervention and OTF‐W incidence in Somerset (IRR = 1.00; 95% CI: 0.80, 1.26; *p* = .976). Further investigation of this comparison area could not identify any major differences in the data available that might explain this effect, and this area was retained in the central area model.

The sensitivity analysis of explanatory variables demonstrated that distance to intervention had a strong influence on the association between the intervention and OTF‐W incidence in the central area of Somerset (Table A1 in Appendix 1). When the distance‐to‐intervention variable was removed from the model, the IRR for the association between the intervention and OTF‐W incidence in Somerset increased and was no longer statistically significant (*p* = .476). This indicated that after adjusting for the other explanatory variables, inclusion of the distance between the intervention and comparison areas variable had an influential effect on the estimated IRR.

### 3.3 Analysis of the impact of the intervention on OTF‐W incidence in surrounding areas

For the buffer areas (Model B), the proportion of the area classed as urban and the proportion of farms with more than one fragment of land in the area were included alongside the a priori explanatory variables as they affected the association between the intervention and OTF‐W incidence in Somerset and reduced the AIC (Table 4). An increased estimated IRR was observed for the buffer area around the intervention area in Somerset (IRR = 1.38; 95% CI: 1.09, 1.75; *p* = .008) while no significant association between the intervention and OTF‐W incidence was observed in the Gloucestershire buffer, having adjusted for explanatory variables (IRR = 0.91; 95% CI: 0.77, 1.07; *p* = .243). Increases in time at risk and historic incidence were associated with an increased OTF‐W incidence in the first 2 years of culling. Associations with an increase in OTF‐W incidence in the intervention areas were also observed for the proportion of herds that were dairy, the proportion of land classed as urban, and the proportion of farms with more than one fragment of land in the area. An increase in the proportion of RBCT proactive land was associated with a decreased OTF‐W incidence in the first 2 years of culling. The Deviance and Pearson goodness‐of‐fit tests for overdispersion (*p* = .762 and *p* = .774, respectively) indicated no evidence of there being significantly more variation in the data than expected.

The sensitivity analysis of OTF‐W incidence in the buffer areas showed that removing Somerset comparison buffer areas WS02B or WS07B affected the estimated association between OTF‐W incidence in the Somerset buffer and comparison area buffers (when WS02B removed: IRR = 1.64, *p* = .181; when WS07B removed: IRR = 1.24, *p* = .125). The removal of WS07B reduced the estimated IRR for intervention in the Gloucestershire buffer area to 0.81 (*p* = .011). However, further examination of these areas could not identify any major differences in the data available that might explain these effects and the comparison areas were retained in the final model.

The sensitivity analysis of explanatory variables demonstrated that the proportion of the herds that were classed as dairy and the proportion of farms with more than one fragment of land in the area appeared to have a strong influence on the association between the intervention and OTF‐W incidence in both intervention buffer areas (Table A2 in Appendix 1). For both areas, the inclusion of these variables increased the estimated IRR. In the Somerset intervention buffer area, this resulted in a significant association between the intervention and OTF‐W incidence. In the Gloucestershire intervention buffer area, adjusting for these factors removed the observed association between the intervention and OTF‐W incidence. Other factors that had an influential effect on the IRR in the Somerset intervention buffer area were median herd size, and the proportion of land that was exposed to proactive culling in the RBCT.

## 4 DISCUSSION

Industry‐led badger culling in England is a component of the current multifaceted bovine TB control policy, not a scientific trial, and as such lacks randomization and other controls. Nonetheless, it is important to monitor the effects of the industry‐led culling and evaluate the impacts taking study limitations into account. The external validity (i.e., the generalizability) of the findings will be limited by the way in which the study areas were selected, and the extent to which the circumstances under which the policy is operating are similar to circumstances where culling is proposed in the future.

### 4.1 Suitability of selected comparison areas

In order to assess the impact of the intervention on cattle TB incidence, we first had to develop a method for selecting suitable comparison areas. For the RBCT, areas were prospectively selected in regions similar in terms of location and geographical characteristics and where the incidence of cattle TB in England was highest (Independent Scientific Group on Cattle TB 2007). RBCT areas were randomized to receive proactive or reactive culling or no culling (survey only). As randomization was not possible with the current policy, comparison areas were matched to purposively selected intervention areas on factors associated with cattle TB incidence including distance between areas and historic OTF‐W incidence. Herd size was used as a criterion because it has been consistently and positively associated with TB in cattle herds (Conlan et al., 2012; Goodchild & Clifton‐Hadley, 2001; Ramirez‐Villaescusa, Medley, Mason, & Green, 2010). The proportion of land previously in an RBCT proactive culling area was also used to match areas, whereas land previously part of an RBCT reactive area was not. This is because no long‐term effect from reactive culling on recurrence of cattle TB incidents has been detected (Karolemeas et al., 2012). Reductions in cattle TB incidents in proactively culled RBCT trial areas were measurable during culling and after the cessation of culling (Donnelly et al., 2007; Jenkins, Woodroffe, & Donnelly, 2010). Further exploratory analyses are consistent with an ongoing, but diminishing (test for temporal trend *p* = .008), benefit of proactive culling at 55–60 months post‐trial (Donnelly, Jenkins, & Woodroffe, 2011).

The method used to select comparison areas maximized the probability of identifying areas that were very similar to the intervention areas in terms of risk factors for TB, thereby reducing the impact of confounding factors in descriptive comparisons of rates. Nevertheless, the matching was not perfect and matching variables were incorporated into the regression models as a priori confounders in an attempt to account for the imperfect matching.

### 4.2 Impact of the intervention on OTF‐W incidence

In annually published surveillance reports describing the cull areas (APHA 2015, 2016), observed OTF‐W incidence was reported for the intervention areas combined to maximize the sample size. In the analyses reported here, a difference in effects was observed between the Somerset intervention area and the Gloucestershire intervention area, also demonstrated in a statistically significant area interaction term in the modeling, so results have been reported separately for the individual areas.

When the effect of intervention in each area was analyzed with adjustment for potentially confounding factors, significantly lower IRRs for intervention were revealed in the Gloucestershire central area in Year 1 (Table A5), and in each of the central areas in Year 2 (Table A6) and when both Year 1 and Year 2 were analyzed together (Table A4). Additional modeling to better understand interarea comparison indicated that OTF‐W incidence was lower in the Gloucestershire intervention area than its comparison areas before culling began (Table S4), as was also observed in the unadjusted analysis. Similar analyses of TB incidence prior to culling were not performed in the RBCT because the randomized selection of areas in that trial was expected to have removed any biases related to differences between areas.

### 4.3 Impact of the intervention on OTF‐W incidence in surrounding areas

The multivariable analysis revealed an increased IRR for the intervention in the Somerset buffer area (IRR: 1.38, *p* = .008) and no effect in the Gloucestershire buffer area (IRR: 0.91, *p* = .243). The observed effect in the Somerset buffer area is consistent with the results of the RBCT, which indicated that an increase in cattle TB incidence could be expected in the buffer areas due to perturbation of the badger population (Donnelly et al., 2006; Woodroffe et al., 2006). When the individual years were investigated (Tables A5 and A6 in Appendix 2), the increased IRR for intervention in the Somerset buffer area was only observed in Year 1, and a lower IRR for intervention in the Gloucestershire buffer area was observed in Year 1 and Year 2 individually. None of the differences in the buffer areas were observed when the year prior to the culls was analyzed (Table A4).

### 4.4 Reliability of the models

The associations between the intervention and OTF‐W incidence in Somerset that were observed in the models including all associated covariates (Table 4) were not observed in the simpler RBCT‐like models which included a small number of covariates (Table A3). The modeling performed for the RBCT did not include additional explanatory factors as differences in these potential confounders between areas were assumed to have been accounted for by the randomization. Indeed, there was no substantial over dispersion despite the limited number of explanatory variables included (Donnelly et al., 2003, 2006). In this study, where randomization was not possible, simpler models that do not control for potential confounding factors may be less effective in detecting an association between industry‐led badger culling and OTF‐W incidence.

The sensitivity analysis performed on the buffer area model identified two Somerset comparison buffer areas as being particularly influential. There was more variation in incidence among the Somerset buffer comparison areas, which might explain why some areas are more influential than others in the models. The greater variation in incidence between buffer areas is not surprising as they are geographically smaller than the central areas and contain fewer herds. The positive influence of the proportion dairy variable and the proportion of farms with more than one fragment of land on the association between culling and OTF‐W incidence in both intervention buffer areas is not unexpected as both are factors that have been found to be positively correlated with incidence in previous studies (Broughan et al., 2016; Goodchild & Clifton‐Hadley, 2001; Porphyre, Stevenson, & McKenzie, 2008). The sensitivity analysis demonstrates that there is considerable uncertainty around the estimates that cannot currently be explained. Our analysis will be subject to confounding in common with all observational studies despite attempts to control for these effects. Further follow‐up and inclusion of data from additional culled areas will likely make future analyses more informative (Donnelly, Bento, Goodchild, & Downs, 2015).

### 4.5 OTF‐W incidents versus all TB incidents

OTF‐W incidents were used as the outcome measure rather than all TB incidents because the RBCT only showed an association between OTF‐W incidence and culling (Independent Scientific Group on Cattle TB, 2007). This reasoning assumes that the current case definition of OTF‐W herds is comparable to the definition of a confirmed incident used during the RBCT. This may not be the case due to operational and policy changes in the management of TB incidents, particularly since the introduction of the TB eradication strategy in 2014 (Defra 2014b). It is not understood why the effects of culling observed in the RBCT were only observed for OTF‐W‐like incidents. To investigate further, we extended our analysis of industry‐led culling to include all TB incidents (Table A7 in Appendix 3). Qualitatively similar results were obtained to those obtained when modeling only OTF‐W incidents, although the estimated effects were slightly weaker. This might suggest that culling has more influence on OTF‐W incidents than those without postmortem evidence of infection, suggesting that there is a stronger association between badgers and the occurrence of OTF‐W incidents compared with all TB incidents. The probability of *M. bovis* infection in a herd will be higher for OTF‐W incidents than for TB incidents that have not been confirmed by postmortem evidence of infection which could possibly increase the power to detect effects (De la Rua‐Domenech et al., 2006).

### 4.6 Difficulties with assessing the impact of industry‐led culls

In Year 1, the target of reducing the estimated precull population by at least 70% was not achieved, nor was spatial coverage of the culling homogeneous (Independent Expert Panel 2014). In Year 2, the minimum number of badgers to be removed was achieved in the Somerset intervention area, but not in the Gloucestershire area (Defra, 2014c). This means culling may not have been as effective as that conducted in the RBCT and could potentially have had an adverse effect on cattle TB if it led to greater badger movement and transmission of *M. bovis*. Furthermore, differences in the implementation of the culls between the two intervention areas due to factors such as protestor activity, noncompliant land holding, and heterogeneous coverage of land, may have resulted in differential effects between areas (Independent Expert Panel 2014).

Changes in bovine TB control policy over the duration of the culls, particularly where its application is not equally distributed between the intervention and comparison areas, will impact the quality of comparisons. The “intervention” in Year 1 consisted of culling alone, whereas in Year 2, it comprised culling and the provision of farm‐level risk management advice. Because the risk management program may have a beneficial effect in reducing transmission of infection, any positive or negative effects on cattle TB incidents detected in Year 2 and subsequent years may be attributable to a combination of policies and not to badger culling alone.

It has been estimated that in order to have sufficient power to be confident of observing significant differences in the incidence of OTF‐W herd incidents, matched intervention and comparison areas will need to be observed for at least 3 years after culling begins, and that this increases to 4 years if only two intervention areas are licensed (Donnelly et al., 2015). As such, it was not expected that significant differences would be observed in the first 2 years of follow‐up, particularly since the proportion of badgers removed was estimated to be lower than in the RBCT. While it is possible that the significant differences observed could be true differences, selection bias cannot be ruled out due to the nonrandomized selection of the cull areas. It is also possible (although unlikely given the low *p*‐values) that the observed effects are due to chance. Caution should always be applied in the interpretation of models which attempt to fit a relatively large number of parameters (Babyak, 2004; Green 1991), particularly when the dataset is small. However, these findings are coherent with findings from the RBCT.

With the limited data available to date, it is possible that spurious associations could have arisen. For example, the central area model for the first 2 years since culling began identified estimated badger sett density as being negatively associated with OTF‐W incidence in the area. This counterintuitive result at first sight casts doubt on the model, but an increase in estimated badger sett density could increase the likelihood that the transmission of *M. bovis* to cattle is from badgers, and thus, culling badgers may have more of an impact on cattle TB incidence. The impact of the efficacy of the culls could not be investigated in this analysis because of the uncertainty around the estimates of badger population reduction (AHVLA 2014).

A number of potential confounding factors have been included in this analysis. However, even with the inclusion of this additional information, it is unlikely that all sources of bias have been estimated or detected. For example, we do not have all the information that may have been considered by groups of farmers and land owners selecting the culling areas. Factors that affect cattle TB risk may also change with time. Furthermore, farmers and landowners aware of the intervention may change their behaviors in ways that affect cattle TB incidence independent of any changes in Government policy (Gale, 2004).

## 5 CONCLUSIONS

We have identified reductions in OTF‐W incidence in both the Somerset and Gloucestershire intervention areas and an increase in OTF‐W incidence in the Somerset buffer area in the first 2 years of industry‐led culls. As this analysis has been performed on only two intervention areas with only 2 years of follow‐up data, and the biological cause–effect relationship behind the statistical associations observed is difficult to determine, it would be unwise to use the findings of this analysis to develop generalizable inferences about the effectiveness of the policy at present. These findings are consistent with findings from the RBCT, although a time lag of around 4 years was observed between culling in the RBCT and measureable significant effects on cattle incidence (Donnelly et al., 2007).

Although a trial randomizing culling to different matched areas would be the most rigorous design for the evaluation of the effect of a badger culling policy, this type of design is not possible when the areas where culling is conducted are selected by stakeholders. The long‐term value of monitoring information from industry‐led culling will depend on the conduct of the culls, the number of areas eventually licensed and the extent to which other bovine TB control policies remain stable. Continued delivery of the intervention in these areas, and further roll out of the intervention to other areas may enable better assessments to be made of the impact of industry‐led culling on TB incidence in cattle.

## CONFLICT OF INTEREST

None declared.

## APPENDIX 1

### Sensitivity analysis of individual explanatory variables

#### 1

**Table A1:** Model estimates for intervention (industry‐led badger culling) in the Somerset and Gloucestershire *central areas in the first 2 years since culling began* when individual explanatory factors are either added (in yellow) or removed (in blue) from three models: a simple RBCT‐like model, the baseline model which was the RBCT‐like model plus factors considered important a priori, and the final model which was the baseline model plus additional explanatory factors. Where there are notable changes in associations from the starting models, the p values are highlighted in bold font. (Final model reported in Table 4 of main body of paper)

		RBCT‐like model			Baseline model			Final model		
		IRR^a^	95% CI	*p* value	IRR^a^	95% CI	*p* value	IRR^a^	95% CI	*p* value
*RBCT factors*: Area, herd years at risk, historic 3 year incidence	Somerset	1.012	0.879–1.165	.868	0.892	0.716–1.112	.310	0.791	0.716–0.874	<.001
	Gloucestershire	0.685	0.577–0.813	<.001	0.606	0.518–0.708	<.001	0.417	0.344–0.507	<.001
Median herd size	Somerset	1.008	0.868–1.170	.922	0.901	0.734–1.106	.319	0.804	0.732–0.884	<.001
	Gloucestershire	0.677	0.580–0.791	<.001	0.620	0.534–0.720	<.001	0.438	0.358–0.537	<.001
Proportion dairy	Somerset	1.174	0.898–1.534	.241	0.798	0.612–1.040	.095	0.710	0.588–0.858	<.001
	Gloucestershire	0.668	0.588–0.760	<.001	0.592	0.470–0.745	<.001	0.408	0.305–0.547	<.001
Distance to intervention	Somerset	0.824	0.663–1.026	.083	1.053	0.923–1.200	.442	0.957	0.847–1.081	**.476**
	Gloucestershire	0.616	0.507–0.749	<.001	0.690	0.639–0.746	<.001	0.508	0.417–0.619	<.001
Badger sett density	Somerset	0.976	0.860–1.107	.705	0.933	0.692–1.259	.652	0.791	0.684–0.915	.002
	Gloucestershire	0.688	0.580–0.816	<.001	0.580	0.483–0.695	<.001	0.390	0.324–0.470	<.001
Proportion RBCT	Somerset	1.004	0.865–1.165	.960	0.892	0.714–1.114	.312	0.790	0.714–0.875	<.001
	Gloucestershire	0.693	0.569–0.845	<.001	0.593	0.508–0.692	<.001	0.416	0.347–0.498	<.001
Total badgers removed historically	Somerset	0.977	0.869–1.099	.701	0.840	0.733–0.962	**.012**	0.848	0.728–0.988	.034
	Gloucestershire	0.622	0.501–0.774	<.001	0.511	0.415–0.631	<.001	0.514	0.442–0.598	<.001
Proportion fragmented	Somerset	0.974	0.845–1.121	.710	0.848	0.727–0.988	**.034**	0.840	0.733–0.962	.012
	Gloucestershire	0.658	0.530–0.816	<.001	0.514	0.442–0.598	<.001	0.511	0.415–0.631	<.001

a Incidence rate ratio.

##### 1.1

**Table A2:** Model estimates for intervention (industry‐led badger culling) in the Somerset and Gloucestershire *buffer areas in the first 2 years since culling began* when individual explanatory factors are either added (in yellow) or removed (in blue) from three models: a simple RBCT‐like model, the baseline model which was the RBCT‐like model plus factors considered important a priori, and the final model which was the baseline model plus additional explanatory factors. Where there are notable changes in associations from the starting models, the p values are highlighted in bold font. (Final model reported in Table 4 of main body of paper)

		RBCT‐like model			Baseline model			Final model		
		IRR^a^	95% CI	*p* value	IRR^a^	95% CI	*p* value	IRR^a^	95% CI	*p* value
*RBCT factors*: Area, herd years at risk, historic 3 year incidence	Somerset	0.818	0.638–1.047	.111	1.177	0.893–1.550	.247	1.379	1.090–1.746	.008
	Gloucestershire	0.998	0.758–1.313	.987	0.992	0.744–1.323	.957	0.908	0.771–1.068	.243
Median herd size	Somerset	0.892	0.689–1.154	.385	1.122	0.799–1.575	.508	1.329	0.989–1.785	**.059**
	Gloucestershire	0.993	0.755–1.307	.961	0.987	0.748–1.302	.926	0.902	0.768–1.059	.207
Proportion dairy	Somerset	1.326	0.921–1.909	.129	0.843	0.565–1.257	.402	0.962	0.712–1.300	**.803**
	Gloucestershire	0.939	0.751–1.173	.577	0.919	0.683–1.236	.577	0.782	0.653–0.936	**.007**
Distance to intervention	Somerset	0.703	0.476–1.039	.077	1.248	0.969–1.608	.086	1.610	1.257–2.072	<.001
	Gloucestershire	0.937	0.701–1.252	.661	1.012	0.786–1.303	.926	0.965	0.849–1.096	.583
Badger sett density	Somerset	0.917	0.646–1.304	.627	1.307	0.929–1.838	.124	1.402	1.060–1.855	.018
	Gloucestershire	0.939	0.715–1.234	.653	0.923	0.706–1.206	.556	0.891	0.765–1.038	.139
Proportion RBCT	Somerset	0.813	0.636–1.040	.099	1.182	0.910–1.535	.211	1.083	0.778–1.508	**.637**
	Gloucestershire	1.024	0.746–1.407	.882	0.982	0.738–1.306	.899	0.937	0.778–1.129	.495
Proportion urban	Somerset	0.984	0.814–1.191	.872	0.758	0.443–1.295	.310	2.193	1.456–3.303	<.001
	Gloucestershire	0.890	0.788–1.005	.060	0.779	0.585–1.036	.086	1.171	0.905–1.515	.230
Proportion fragmented	Somerset	0.819	0.645–1.041	.103	2.193	1.456–3.303	**<.001**	0.758	0.443–1.295	**.310**
	Gloucestershire	1.019	0.719–1.444	.917	1.171	0.905–1.515	.230	0.779	0.585–1.036	**.086**

a Incidence rate ratio.

## APPENDIX 2

### ADDITIONAL MODEL VALIDATION

#### Replicating the RBCT models

In order to compare the current results with those of the RBCT, we assessed the effect of including just those factors that had been identified as important in the RBCT modeling when analyzing the first 2 years of culling (Table A3). As for the larger models, the inclusion of an interaction between the intervention and area was assessed. The interaction generated a *p*‐value of .005 for the central areas and .399 for the buffer areas. This indicated that the effects of the interventions in the two central areas should be examined separately. When only time at risk (a measure comparable in effect to the baseline number of herds included in the RBCT analyses), historic incidence, area, and intervention (badger culling) were included in the models the AIC was poorer (larger) compared with the larger models. In this model, significantly lower incidence was still observed in the Gloucestershire central area (IRR = 0.69; *p* < .001) but there was no significant effect of culling in the Somerset central area or either of the buffer areas.

##### 1

**Table A3:** Multivariable Poisson regression models describing the effect of intervention separated by area on the number of OTF‐W^b^ incidents *in the first 2 years since culling began*, adjusted for a reduced set of explanatory variables as applied in analysis of the RBCT (Donnelly et al., 2003, 2006). Model 1 describes the effect in the central intervention areas compared with matched central comparison areas, and Model 2 describes the effect in the 2‐km buffer area for the intervention areas compared with buffer areas for matched comparison areas

	IRR^a^	Robust *SE*	*p* value	95% Confidence interval	
Model 1—Central areas					
Intervention effect in Somerset area^b^	1.012	0.073	.868	0.879	1.165
Intervention effect in Gloucestershire area^b^	0.685	0.060	<.001	0.577	0.813
Area = Somerset	1.008	0.082	.926	0.859	1.182
Log transformed herd years at risk in the first 2 years of culling	5.436	1.284	<.001	3.421	8.635
Log transformed OTF‐W incidence rate over all 3 years prior	2.075	0.143	<.001	1.813	2.374
Model 2—Buffer areas					
Intervention effect in Somerset buffer area^b^	0.818	0.103	.111	0.638	1.047
Intervention effect in Gloucestershire buffer area^b^	0.998	0.140	.987	0.758	1.313
Area = Somerset	1.242	0.209	.199	0.892	1.728
Log transformed herd years at risk in the first 2 years of culling	2.259	0.567	.001	1.381	3.696
Log transformed OTF‐W incidence rate over all 3 years prior	1.588	0.134	<.001	1.346	1.874

Officially Tuberculosis Free status Withdrawn.

a Incidence rate ratio.

b Intervention is industry‐led badger culling, as described in the Section 1.

#### Statistical analysis of the year prior to the culls

In the initial models for the year prior to the culls which contained a single intervention variable and an area variable, the inclusion of an interaction between the intervention and area generated a *p*‐value of <.001 for the central areas and *p* = .775 for the buffer areas, so the effects were examined separately for each area.

For the central areas (Table A4, Model 1), the number of badgers removed historically was included in the model alongside the a priori explanatory variables as it reduced the AIC. Despite no “intervention” actually taking place, a significantly lower IRR for intervention in the Gloucestershire central area was observed (IRR = 0.59, *p* = .002), while no significant association with the intervention was observed in the Somerset central area (IRR = 1.05, *p* = .521). Increases in time at risk and historic incidence were significantly associated with an increase in OTF‐W incidence in the intervention areas. The Deviance and Pearson goodness‐of‐fit tests for overdispersion generated large *p* values (*p* = .973 for both tests) indicating that there was no evidence of there being significantly more variation in the data than expected.

For the buffer areas (Table A4, Model 2), the length of motorway through an area was included in the model alongside the a priori explanatory variables as it modified the coefficient for the association between the intervention and OTF‐W incidence in the Gloucestershire buffer area. There appeared to be no effect of intervention on OTF‐W incidence in the buffer areas, having adjusted for explanatory variables. Increases in time at risk, historic two‐year incidence rate and median herd size were significantly associated with an increase in the numbers of OTF‐W incidents in the year prior to the culls. An increase in the estimated number of badger setts per 100 km^2^ was associated with a decrease in OTF‐W incidence in the year prior to the culls. The Deviance and Pearson goodness‐of‐fit tests for overdispersion generated large *p* values (*p* = .592 and *p* = .593, respectively) indicating that there was no evidence of there being significantly more variation in the data than expected.

**Table A4:** Multivariable Poisson regression models describing the effect of intervention (badger culling) stratified by area on the number of OTF‐W^a^ incidents *in the year prior to the culls*, adjusted for explanatory variables. Model 1 describes the effect in the central intervention areas compared with matched central comparison areas, and Model 2 describes the effect in the 2‐km buffer area for the intervention areas compared with buffer areas for matched comparison areas

	IRR^b^	Robust *SE*	*p* value	95% Confidence interval	
Model 1—Central areas					
Intervention effect in Somerset area^c^	1.049	0.078	.521	0.907	1.212
Intervention effect in Gloucestershire area^c^	0.588	0.100	.002	0.422	0.821
Area = Somerset	1.002	0.139	.986	0.764	1.315
Log transformed herd years at risk in year prior to culls	1.642	0.337	.016	1.098	2.454
Log transformed OTF‐W incidence rate over 2 years prior	1.917	0.258	<.001	1.472	2.496
Log transformed median herd size	1.482	0.798	.465	0.516	4.260
Proportion of herds that are dairy	1.004	0.003	.229	0.997	1.011
Distance to intervention (km)	0.999	0.001	.635	0.997	1.002
Estimated badger sett density per 100 km^2^	1.000	0.005	.924	0.991	1.010
Proportion of land involved in proactive culling in the RBCT	0.948	0.035	.145	0.882	1.019
Log transformed number of badgers culled historically	0.990	0.010	.316	0.971	1.010
Model 2—Buffer areas					
Intervention effect in Somerset area^c^	1.036	0.129	.777	0.811	1.322
Intervention effect in Gloucestershire area^c^	1.097	0.212	.632	0.751	1.603
Area = Somerset	0.934	0.083	.444	0.785	1.112
Log transformed herd years at risk in year prior to culls	1.989	0.211	<.001	1.615	2.449
Log transformed OTF‐W incidence rate over 2 years prior	1.702	0.185	<.001	1.376	2.105
Log transformed median herd size	1.626	0.318	.013	1.108	2.386
Proportion of herds that are dairy	1.000	0.005	.969	0.991	1.010
Distance to intervention (km)	1.003	0.002	.149	0.999	1.006
Estimated badger sett density per 100 km^2^	0.271	0.138	.011	0.100	0.737
Proportion of land involved in proactive culling in the RBCT	1.007	0.005	.185	0.997	1.018
Length of motorway in the area	1.005	0.006	.422	0.993	1.017

a Officially Tuberculosis Free status Withdrawn.

b Incidence rate ratio.

c Intervention is industry‐led badger culling, as described in the Section 1.

#### Statistical analysis of each of the first two individual years after culling began

In addition to examining the association between the intervention and OTF‐W incidence in the 2 years of culling, we also examined the association for each of the individual years after culling took place (Year 1 and Year 2). In Year 1, a significant association between the intervention and OTF‐W incidence was only observed for the Gloucestershire central area (IRR = 0.56, *p* = .001), while significant associations between the intervention and OTF‐W incidence were observed in both the buffer areas, although the direction of the effect differed (Somerset – IRR = 1.89, *p* < .001; Gloucestershire – 0.75, *p* = .016) (Table A5). In Year 2, a significant association between the intervention and OTF‐W incidence was observed for both central areas characterized by a lower incidence in both areas (Somerset – IRR = 0.67, *p* < .001; Gloucestershire – 0.62, *p* = .049), while a significant association between the intervention and OTF‐W incidence was only observed in the Gloucestershire buffer area (IRR = 0.72, *p* = .038).

**Table A5:** Multivariable Poisson regression models describing the effect of intervention (badger culling) stratified by area on the number of OTF‐W^a^ incidents *in the first year since culling began*, adjusted for explanatory variables. Model 1 describes the effect in the central intervention areas compared with matched central comparison areas, and Model 2 describes the effect in the 2‐km buffer area for the intervention areas compared with buffer areas for matched comparison areas

	IRR^b^	*SE*	*p* value	95% Confidence interval	
Model 1—Central areas					
Intervention effect in Somerset area^c^	0.908	0.158	.580	0.647	1.276
Intervention effect in Gloucestershire area^c^	0.563	0.099	.001	0.399	0.796
Area = Somerset	0.964	0.186	.851	0.660	1.408
Log transformed herd years at risk in year prior to culls	3.716	1.251	<.001	1.920	7.189
Log transformed OTF‐W incidence rate over 2 years prior	2.311	0.616	.002	1.371	3.896
Log transformed median herd size	0.411	0.300	.223	0.098	1.718
Proportion of herds that are dairy	1.021	0.008	.006	1.006	1.037
Distance to intervention (km)	0.996	0.002	.060	0.993	1.000
Estimated badger sett density per 100 km^2^	0.989	0.007	.140	0.976	1.004
Proportion of land involved in proactive culling in the RBCT	0.963	0.084	.667	0.812	1.143
Model 2—Buffer areas					
Intervention effect in Somerset area^c^	1.886	0.182	<.001	1.561	2.279
Intervention effect in Gloucestershire area^c^	0.748	0.090	.016	0.591	0.948
Area = Somerset	0.805	0.052	.001	0.710	0.914
Log transformed herd years at risk in year prior to culls	1.688	0.112	<.001	1.482	1.921
Log transformed OTF‐W incidence rate over 2 years prior	2.136	0.132	<.001	1.893	2.411
Log transformed median herd size	1.547	0.157	<.001	1.268	1.888
Proportion of herds that are dairy	1.041	0.005	<.001	1.031	1.052
Distance to intervention (km)	1.003	0.001	.001	1.001	1.004
Estimated badger sett density per 100 km^2^	0.071	0.022	<.001	0.039	0.131
Proportion of land involved in proactive culling in the RBCT	0.973	0.007	<.001	0.961	0.986
Length of motorway in the area (km)	1.021	0.004	<.001	1.013	1.029
Proportion of farms with more than one fragment of land in the area	1.054	0.006	<.001	1.042	1.066

a Officially Tuberculosis Free status Withdrawn.

b Incidence rate ratio.

c Intervention is industry‐led badger culling, as described in the Section 1.

**Table A6:** Multivariable Poisson regression models describing the effect of intervention (badger culling) stratified by area on the number of OTF‐W^a^ incidents *in the second year since culling began*, adjusted for explanatory variables. Model 1 describes the effect in the central intervention areas compared with matched central comparison areas, and Model 2 describes the effect in the 2‐km buffer area for the intervention areas compared with buffer areas for matched comparison areas

	IRR^b^	*SE*	*p* value	95% Confidence interval	
Model 1—Central areas					
Intervention effect in Somerset area^c^	0.668	0.042	<.001	0.591	0.755
Intervention effect in Gloucestershire area^c^	0.615	0.152	.049	0.379	0.999
Area = Somerset	1.291	0.317	.297	0.798	2.089
Log transformed herd years at risk in year prior to culls	3.467	0.641	<.001	2.413	4.981
Log transformed OTF‐W incidence rate over 2 years prior	1.126	0.182	.462	0.820	1.547
Log transformed median herd size	1.844	1.635	.490	0.324	10.483
Proportion of herds that are dairy	1.001	0.005	.809	0.992	1.010
Distance to intervention (km)	0.995	0.001	<.001	0.994	0.997
Estimated badger sett density per 100 km^2^	1.000	0.003	.985	0.994	1.006
Proportion of land involved in proactive culling in the RBCT	0.993	0.047	.889	0.905	1.090
Log transformed number of badgers culled historically	1.052	0.023	.021	1.008	1.099
Proportion of land classed as urban	1.062	0.019	.001	1.026	1.099
Proportion of farms with more than one fragment of land in the area	1.043	0.010	<.001	1.025	1.063
Model 2—Buffer areas					
Intervention effect in Somerset area^c^	1.278	0.443	.478	0.649	2.520
Intervention effect in Gloucestershire area^c^	0.717	0.115	.038	0.523	0.982
Area = Somerset	1.350	0.411	.324	0.743	2.452
Log transformed herd years at risk in year prior to culls	1.753	0.288	.001	1.270	2.419
Log transformed OTF‐W incidence rate over 2 years prior	1.812	0.412	.009	1.161	2.830
Log transformed median herd size	1.005	0.334	.987	0.525	1.927
Proportion of herds that are dairy	1.008	0.017	.618	0.976	1.042
Distance to intervention (km)	0.996	0.003	.155	0.991	1.001
Estimated badger sett density per 100 km^2^	6.724	8.506	.132	0.563	80.248
Proportion of land involved in proactive culling in the RBCT	0.989	0.013	.408	0.964	1.015
Proportion of land classed as urban	1.075	0.028	.006	1.021	1.132
Proportion of farms with more than one fragment of land in the area	1.046	0.021	.024	1.006	1.087

a Officially Tuberculosis Free status Withdrawn.

b Incidence rate ratio.

c Intervention is industry‐led badger culling, as described in the Section 1.

## APPENDIX 3

### Analysis of all cattle TB incidents

**Table A7:** Multivariable Poisson regression models describing the effect of intervention (badger culling) stratified by area on the number of TB incidents *in the first 2 years since culling began*, adjusted for explanatory variables. Model 1 describes the effect in the central intervention areas compared with matched central comparison areas, and Model 2 describes the effect in the 2‐km buffer area for the intervention areas compared with buffer areas for matched comparison areas

	IRR^a^	Robust *SE*	*p* value	95% Confidence interval	
Model 1—Central areas					
Intervention effect in Somerset area^b^	0.886	0.050	.033	0.793	0.991
Intervention effect in Gloucestershire area^b^	0.586	0.040	<.001	0.513	0.670
Area = Somerset	1.160	0.102	.090	0.977	1.377
Log transformed herd years at risk in year prior to culls	4.038	0.701	<.001	2.873	5.674
Log transformed OTF‐W incidence rate over 2 years prior	1.476	0.138	<.001	1.229	1.773
Log transformed median herd size	0.218	0.101	.001	0.088	0.540
Proportion of herds that are dairy	1.018	0.004	<.001	1.011	1.025
Distance to intervention (km)	0.998	0.001	.092	0.997	1.000
Estimated badger sett density per 100 km^2^	0.994	0.003	.045	0.989	1.000
Proportion of land involved in proactive culling in the RBCT	1.016	0.024	.503	0.970	1.065
Proportion of farms with more than one fragment of land in the area	1.021	0.006	<.001	1.010	1.032
Model 2—Buffer areas					
Intervention effect in Somerset area^b^	1.333	0.199	.054	0.994	1.788
Intervention effect in Gloucestershire area^b^	0.902	0.094	.319	0.736	1.105
Area = Somerset	1.118	0.129	.334	0.892	1.401
Log transformed herd years at risk in year prior to culls	1.880	0.201	<.001	1.524	2.319
Log transformed OTF‐W incidence rate over 2 years prior	1.821	0.186	<.001	1.491	2.224
Log transformed median herd size	1.044	0.163	.783	0.769	1.417
Proportion of herds that are dairy	1.023	0.010	.025	1.003	1.043
Distance to intervention (km)	0.998	0.002	.283	0.995	1.002
Estimated badger sett density per 100 km^2^	1.337	0.770	.614	0.432	4.136
Proportion of land involved in proactive culling in the RBCT	0.982	0.006	.003	0.970	0.994
Proportion of land classed as urban	1.043	0.014	.002	1.016	1.072
Proportion of farms with more than one fragment of land in the area	1.033	0.010	.001	1.014	1.052

a Incidence rate ratio.

b Intervention is industry‐led badger culling, as described in the Section 1.

## References

[ece33254-bib-0001] AHVLA (2014). Monitoring the efficacy of badger population reduction by controlled shooting during the first six weeks of the pilots. Report to Defra 31st January 2014. Retrieved from https://www.gov.uk/government/uploads/system/uploads/attachment_data/file/300383/ahvla-efficacy-report.pdf. Accessed July 13, 2015.

[ece33254-bib-0002] APHA (2015). Bovine tuberculosis: Infection status in cattle in England. Annual surveillance report for the period January to December 2014. Retrieved from https://www.gov.uk/government/uploads/system/uploads/attachment_data/file/467803/england-surveillance-report14.pdf

[ece33254-bib-0003] APHA (2016). Report of the incidence of bovine tuberculosis in cattle in 2014 – 2015 in the areas of Somerset and Gloucestershire exposed to two years of industry‐led badger control. Retrieved from https://www.gov.uk/government/uploads/system/uploads/attachment_data/file/548714/tb-badger-control-second-year-analysis.pdf

[ece33254-bib-0004] Babyak, M. (2004). What you see may not be what you get: A brief nontechnical introduction to overfitting in regression‐type models. Psychosomatic Medicine, 66(3), 411–421. 15184705 10.1097/01.psy.0000127692.23278.a9

[ece33254-bib-0005] Broughan, J. M. , Maye, D. , Carmody, P. , Brunton, L. A. , Ashton, A. , Wint, W. , … Enticott, G. (2016). Farm characteristics and farmer perceptions associated with bovine tuberculosis incidents in areas of emerging endemic spread. Preventive Veterinary Medicine, 129, 88–98. 27317326 10.1016/j.prevetmed.2016.05.007

[ece33254-bib-0006] Burnham, K. P. , & Anderson, D. R. (2002). Model selection and Multi‐Model Inference: A practical information‐theoretic approach. New York, NY: Springer‐Verlag.

[ece33254-bib-0008] Conlan, A. J. , McKinley, T. J. , Karolemeas, K. , Pollock, E. B. , Goodchild, A. V. , Mitchell, A. P. , … Wood, J. L. (2012). Estimating the hidden burden of bovine tuberculosis in Great Britain. PLoS Computational Biology, 8(10), e1002730 https://doi.org/10.1371/journal.pcbi.1002730 23093923 10.1371/journal.pcbi.1002730 PMC3475695

[ece33254-bib-0009] De la Rua‐Domenech, R. , Goodchild, A. T. , Vordermeier, H. M. , Hewinson, R. G. , Christiansen, K. H. , & Clifton‐Hadley, R. S. (2006). Ante mortem diagnosis of tuberculosis in cattle: A review of the tuberculin tests, gamma‐interferon assay and other ancillary diagnostic techniques. Research in Veterinary Science, 81(2), 190–210. 16513150 10.1016/j.rvsc.2005.11.005

[ece33254-bib-0010] Defra (2014a). Defra response. Pilot badger culls in Somerset and Gloucestershire: Report by the Independent Expert Panel. Retrieved from https://www.gov.uk/government/uploads/system/uploads/attachment_data/file/300424/pb14158-defra-response-independent-expert-panel.pdf. Accessed April 8, 2014.

[ece33254-bib-0011] Defra (2014b). the strategy for achieving officially bovine tuberculosis free status for England. Retrieved from https://www.gov.uk/government/uploads/system/uploads/attachment_data/file/300447/pb14088-bovine-tb-strategy-140328.pdf. Accessed July 12, 2016.

[ece33254-bib-0012] Defra (2014c). Setting the minimum and maximum numbers for Year 2 of the badger culls. Advice to Natural England. Retrieved from https://www.gov.uk/government/uploads/system/uploads/attachment_data/file/347536/badger-cull-setting-min-max-numbers-2014.pdf. Accessed December 22, 2016.

[ece33254-bib-0013] Defra (2014d). Bovine TB: summary of badger control monitoring during 2014. Retrieved from https://www.gov.uk/government/publications/bovine-tb-summary-of-badger-control-monitoring-during-2014. Accessed July 19, 2016.

[ece33254-bib-0014] Defra (2015). Guidance to Natural England Licences to kill or take badgers for the purpose of preventing the spread of bovine TB under section 10(2)(a) of the Protection of Badgers Act 1992. Retrieved from https://www.gov.uk/government/uploads/system/uploads/attachment_data/file/69464/pb13692-bovinetb-guidance-ne.pdf, Accessed May 2, 2014.

[ece33254-bib-0015] Donnelly, C. A. , Bento, A. I. , Goodchild, A. V. , & Downs, S. H. (2015). Exploration of the power or routine surveillance data to assess the impacts of industry‐led badger culling on bovine tuberculosis incidence in cattle herds. Veterinary Record, 177, 417. 26374782 10.1136/vr.103201 PMC4680152

[ece33254-bib-0016] Donnelly, C. A. , Jenkins, H. , & Woodroffe, R. (2011). Analysis of further data (to 28 August 2011) on the impacts on cattle TB incidence of repeated badger culling. Retrieved from http://journals.plos.org/plosone/article/comment?id=10.1371/annotation/2444ce5c-b56d-4ea3-a554-ffba25152c66

[ece33254-bib-0017] Donnelly, C. A. , Wei, G. , Johnston, W. T. , Cox, D. R. , Woodroffe, R. , Bourne, F. J. , … Morrison, W. I. (2007). Impacts of widespread badger culling on cattle tuberculosis: Concluding analyses from a large‐scale field trial. International Journal of Infectious Diseases, 11(4), 300–308. 17566777 10.1016/j.ijid.2007.04.001

[ece33254-bib-0018] Donnelly, C. A. , Woodroffe, R. , Cox, D. R. , Bourne, F. J. , Cheeseman, C. L. , Clifton‐Hadley, R. S. , … Morrison, W. I. (2006). Positive and negative effects of widespread badger culling on tuberculosis in cattle. Nature, 439, 843–846. 16357869 10.1038/nature04454

[ece33254-bib-0019] Donnelly, C. A. , Woodroffe, R. , Cox, D. R. , Bourne, J. , Gettinby, G. , le Fevre, A. M. , … Morrison, W. I. (2003). Impact of localized badger culling on tuberculosis incidence in British cattle. Nature, 426(6968), 834–837. 14634671 10.1038/nature02192

[ece33254-bib-0020] Downs, S. H. , Clifton‐Hadley, R. S. , Upton, P. U. , Milne, I. , Ely, E. , Gopal, R. , … Sayers, A. R. (2013). Tuberculin manufacturing source and breakdown incidence rate of bovine tuberculosis in British cattle, 2005–2009. Veterinary Record, 172(4), 98. 23355712 10.1136/vr.100679

[ece33254-bib-0021] Gale, E. A. M. (2004). The Hawthorne studies—A fable for our times? QJM: An International Journal of Medicine, 97(7), 439–449. 15208432 10.1093/qjmed/hch070

[ece33254-bib-0022] Goodchild, A. V. , & Clifton‐Hadley, R. S. (2001). Cattle‐to cattle transmission of *Mycobacterium bovis* . Tuberculosis (Edinb), 81(1–2), 23–41. 11463222 10.1054/tube.2000.0256

[ece33254-bib-0023] Green, S. (1991). How many subjects does it take to do a regression analysis? Multivariate Behavioural Research, 26(3), 499–510. 10.1207/s15327906mbr2603_7 26776715

[ece33254-bib-0024] Harris, K. A. , Brunton, L. , Brouwer, A. , Romero Garcia, M. P. , Gibbens, J. C. , Smith, N. H. , & Upton, P. A. (2017). Bovine TB infection in cattle in Great Britain in 2015. Veterinary Record, 180(7), 170–175. 28213421 10.1136/vr.j759

[ece33254-bib-0025] Huber, P. J. (1967). The behavior of maximum likelihood estimates under nonstandard conditions Proceedings of the Fifth Berkeley Symposium on Mathematical Statistics and Probability. Statistics, Vol. 1, 221‐233. Berkeley, Calif: University of California Press.

[ece33254-bib-0026] Independent Expert Panel (2014). Pilot badger culls in Somerset and Gloucestershire. Report by the Independent Expert Panel. Retrieved from https://www.gov.uk/government/uploads/system/uploads/attachment_data/file/300382/independent-expert-panel-report.pdf. Accessed July 22, 2015

[ece33254-bib-0027] Independent Scientific Group on Cattle TB (2007). Bovine TB: The scientific evidence – final report of the independent scientific group on cattle TB. Defra Publications, London HMSO. Retrieved from http://webarchive.nationalarchives.gov.uk/20090330154646/www.defra.gov.uk/animalh/tb/isg/pdf/final_report.pdf. Accessed July 12, 2016.

[ece33254-bib-0028] Jenkins, H. E. , Woodroffe, R. , & Donnelly, C. A. (2010). The duration of the effects of repeated widespread badger culling on cattle tuberculosis following the cessation of culling. PLoS ONE, 5, e9090 https://doi.org/10.1371/journal.pone.0009090 20161769 10.1371/journal.pone.0009090 PMC2818840

[ece33254-bib-0029] Judge, J. , Wilson, G. J. , Macarthur, R. , Delahay, R. J. , & McDonald, R. A. (2014). Density and abundance of badger social groups in England and Wales in 2011–2013. Scientific Reports, 4, 3809. 24457532 10.1038/srep03809 PMC3899851

[ece33254-bib-0030] Karolemeas, K. , Donnelly, C. A. , Conlan, A. J. , Mitchell, A. P. , Clifton‐Hadley, R. S. , … McKinley, T. J. (2012). The effect of badger culling on breakdown prolongation and recurrence of bovine tuberculosis in cattle herds in Great Britain. PLoS ONE, 7(12), e51342. 23236478 10.1371/journal.pone.0051342 PMC3517421

[ece33254-bib-0031] Porphyre, T. , Stevenson, M. A. , & McKenzie, J. (2008). Risk factors for bovine tuberculosis in New Zealand cattle farms and their relationship with possum control strategies. Preventive Veterinary Medicine, 86, 93–106. 18479769 10.1016/j.prevetmed.2008.03.008

[ece33254-bib-0032] Ramirez‐Villaescusa, A. M. , Medley, G. F. , Mason, S. , & Green, L. E. (2010). Risk factors for a herd breakdown with bovine tuberculosis in 148 cattle herds in the southwest of England. Preventive Veterinary Medicine, 95(3–4), 224–230. 20399521 10.1016/j.prevetmed.2010.03.009

[ece33254-bib-0033] White, H. (1982). Maximum likelihood estimation of misspecified models. Econometrica, 50, 1–25.

[ece33254-bib-0035] Woodroffe, R. , Donnelly, C. A. , Cox, D. R. , Bourne, F. J. , Cheeseman, C. L. , Delahay, R. J. , … Morrison, W. I. (2006). Effects of culling on badger *Meles meles* spatial organization: Implications for the control of bovine tuberculosis. Journal of Applied Ecology, 43, 1–10.

